# Cannabis Use Among Lower-Extremity Arthroplasty Patients Does Not Lead to Worse Postoperative Outcomes

**DOI:** 10.7759/cureus.31964

**Published:** 2022-11-28

**Authors:** Viraj Sharma, Logan Laubach, John W Krumme, Jibanananda Satpathy

**Affiliations:** 1 Orthopaedic Surgery, Virginia Commonwealth University School of Medicine, Richmond, USA; 2 Orthopaedic Surgery, University of Missouri Kansas City School of Medicine, Leawood, USA

**Keywords:** revision arthroplasty, promis scores, post-operative complications, cannabis, marijuana, arthroplasty

## Abstract

Introduction: Smoking and general categorizations of substance use are linked with increased postoperative complications following total knee arthroplasty (TKA) and total hip arthroplasty (THA). There is a lack of similar evidence on how cannabis use may affect outcomes after arthroplasty. The present study aims to compare postoperative outcomes in cannabis users versus non-cannabis users who underwent THA/TKA. We hypothesize that cannabis users will have no difference in primarily the complication rate, revision rate, and secondarily post-operative Patient-Reported Outcomes Information System (PROMIS) scores, hospital stay, or pain compared to matched controls.

Methods: Billing codes were used to generate lists of hip/knee arthroplasty patients from 2013 to 2019 at our institution. In the case group, cannabis use was confirmed via chart review. Cannabis-using patients were matched appropriately with non-users by (1) the same arthroplasty procedure; (2) BMI ± 3.5; (3) age ± 3 years; (4) sex. Data on postoperative outcomes were collected from charts and compared between groups using either a Chi-square test for qualitative variables or a paired t-test for quantitative variables.

Results: A total of 24 patients with an average age of 57.1 and a BMI of 30.6 were confirmed to have isolated cannabis use. They were matched to 24 patients with an average age of 57.6 and a BMI of 31.4. There were no significant differences in the complication rate (4.2% vs 4.2%, p=1.00), the revision rate (0% vs 4.2%, p=0.31), days of hospital stay (2.7 vs 3.3, p=0.22), or postoperative pain (4.7 vs 4.9, p=0.86). Similarly, there were no significant differences in all PROMIS score measures.

Discussion/conclusions: Current research shows that cannabis use may lead to increased revision arthroplasty and decreased mortality, with mixed findings regarding post-surgical complications. The present study suggests that cannabis-using patients have no difference in postoperative complication rate, revision rate, PROMIS scores, hospital stay, or pain compared to matched controls.

## Introduction

Cannabis is now legal in 39 states for medically related uses and in 19 states for recreational use, along with the District of Columbia. This widespread legalization has led to an increase in both cannabis use and willingness to admit use to providers, including orthopedic surgeons [1]. The rise in cannabis use among patients may also be escalating due to a significant proportion of patients with musculoskeletal injuries believing in its efficacy in treating pain, though research has produced mixed findings in terms of measures such as opioid medication needs in the postoperative period [2-4].

The widespread use of cannabis among patients, whether for recreational or pain management purposes, becomes an important topic of consideration as postsurgical outcomes in arthroplasty patients can be influenced by drug use [5]. Smoking and general categorizations of substance misuse are linked with increased postoperative complications, mortality, and opioid use following total knee arthroplasty (TKA) and total hip arthroplasty (THA) [6-8]. There is limited evidence on how cannabis use may affect outcomes after surgery, especially in regard to arthroplasty [9-11]. With more patients using cannabis for orthopedic pain management, further research regarding arthroplasty outcomes in cannabis users is needed to better understand the risks and clinical recommendations in this growing population.

The present study aims to compare postoperative outcomes in cannabis users versus non-cannabis users. We hypothesize that cannabis users will have no difference in primarily the complication rate, revision rate, and secondarily post-operative Patient-Reported Outcomes Information System (PROMIS) scores, hospital stay, or pain compared to matched controls.

## Materials and methods

Following IRB approval (HM20022191) at our institution for the study population, our institution’s billing department generated lists of hip/knee arthroplasty patients from January 2013 to January 2019 using CPT codes (27130, 27132, 27125, 27440, 27441, 27442, 27443, 27445, 27446, and 27447). Patients were further subdivided using appropriate ICD-9 (304.30-304.33, 305.20-305.23) or ICD-10 (F12) codes by those with substance abuse or cannabis use.

In the case group, cannabis use was confirmed via chart review. Patients were excluded if they were: (1) under the age of 18; (2) had other confirmed non-cannabis substance abuse within 30 days of surgery; (3) had no follow-up records following surgery. Cannabis-using patients were matched appropriately with non-users by: (1) the same arthroplasty procedure; (2) BMI ± 3.5; (3) age ± 3 years; (4) sex.

Data on postoperative outcomes, including complications, revisions, length of hospital stay, pain scores, and PROMIS scores, were collected from charts. These variables were compared between cannabis users and matched controls using either a Chi-square test for qualitative variables or a paired t-test for quantitative variables on the statistical software JMP Pro (version 16.1.0, JMP Statistical Discovery, Cary, NC).

## Results

A total of 24 patients with an average age of 57.1 and a BMI of 30.6 were confirmed to have isolated cannabis use. They were matched to 24 patients with an average age of 57.6 and a BMI of 31.4. Each group comprised 13 (54.2%) males and 11 (45.8%) females, with 15 (62.5%) patients undergoing total hip arthroplasty and 9 (37.5%) patients undergoing total knee arthroplasty. Indications for surgery in the cannabis and control groups included arthritis [17 (70.8%) vs 19 (79.2%)], osteonecrosis [3 (12.5%) vs 2 (8.3%)], fracture [3 (12.5%) vs 1 (4.2%)], infection [0 vs 2 (8.3%)], and persistent pain [1 (4.2%) vs 0] (Table 1).

**Table 1:** Patient demographics

	Cannabis (N=24)	Control (N=24)
Age	57.1 (SD=9.89)	57.6 (SD=10.2)
BMI	30.6 (SD=6.75)	31.4 (SD=6.49)
Sex		
Male	13 (54.2%)	13 (54.2%)
Female	11 (45.8%)	11 (45.8%)
Surgical indication		
Arthritis	17 (70.8%)	19 (79.2%)
Osteonecrosis	3 (12.5%)	2 (8.3%)
Fracture	3 (12.5%)	1 (4.2%)
Infection	0 (0.0%)	2 (8.3%)
Pain	1 (4.2%)	0 (0.0%)

The complication rate in both groups was equivalent at 4.2% (p=1.00), with one patient in the cannabis group having persistent pain due to postoperative psoas tendinopathy and one patient in the control group having a complicated postoperative course including acute hypoxemic respiratory failure and persistent infection requiring incision and drainage. There was no significant difference in revision rate between groups (0% vs 4.2%, p=0.31), with one patient in the control group requiring revision hip arthroplasty due to recurrent hip instability. There were also no significant differences between the cannabis and control groups in days of hospital stay (2.7 vs 3.3, p=0.22) or postoperative pain rating (4.7 vs 4.9, p=0.86) all PROMIS score measures (Table 2).

**Table 2:** Patient functional outcomes

	Cannabis (N=24)	Control (N=24)	p-value
Hospital stay (days)	2.7 (SD=1.22)	3.3 (SD=1.49)	0.22
Complications	1 (4.2%)	1 (4.2%)	1.00
Revision surgery	0 (0%)	1 (4.2%)	0.31
Pain score (0-10 scale)	4.7 (SD=2.11)	4.9 (SD=2.34)	0.86
PROMIS scores	Cannabis (N=24)	Control (N=24)	p-value
General health	2.8 (SD=0.90)	2.9 (SD=0.97)	0.62
Physical health	2.8 (SD=0.93)	2.5 (SD=0.98)	0.28
Mental health	3.2 (SD=0.82)	3.3 (SD=0.77)	0.53
Social relationships	3.0 (SD=0.93)	2.9 (SD=1.25)	0.88
Physical performance	2.6 (SD=1.34)	2.6 (SD=1.27)	0.90

## Discussion

Current research has mixed findings regarding how cannabis use influences postsurgical outcomes. In regards to revision, one study has shown an increased risk of revision and decreased time to revision after TKA in patients who use cannabis [11], while another study has shown that there were no significant differences in reoperation or hospital readmission in cannabis users following total knee arthroplasty [10]. There has been limited research comparing the rates of hip or knee arthroplasty requiring revision or reoperation in cannabis users, and similar ambiguity has been seen comparing postoperative complications, morbidity, and mortality. The present study suggests that cannabis use does not correlate with an increased risk of revision, postoperative complications, or poorer physical health.

The results in this study also indicate no difference in postoperative complications or physical and mental health between cannabis users and non-users receiving total hip or knee arthroplasty (Table 2). These findings align with current studies showing no beneficial or adverse effects [10], while another study further exhibited decreased mortality in patients using cannabis before and after TKAs, THAs, and traumatic femur fixations [9]. The effects of smoking tobacco in patients receiving TKAs and THAs and worse postoperative outcomes such as increased hospital stays, myocardial infarctions, and overall morbidity and mortality are well documented [6,7,12-15]. The effects of cannabis use are not as clear, and some studies group cannabis use with other substances misused such as opioids, cocaine, amphetamines, and inhalants, and show significantly worse outcomes in these drug abuse groups [8]. Grouping cannabis users with other illicit substance users can make it difficult to discern which postoperative complications are attributable to cannabis alone.

There were no differences in pain scores between cannabis users and control groups. This topic of postoperative pain control in cannabis users after arthroplasty has increased in popularity, with many patients favoring the use of cannabis in place of opiates [1-3]. Cannabis use in the preoperative and postoperative periods following arthroplasty has not been shown to decrease opiate usage, and postoperative opiate requirements are indifferent between cannabis users [4,16,17]. Another study found that cannabis users had higher postoperative pain scores and opiate usage compared to nonusers, while they did not require increased anesthesia or sedation intraoperatively [18]. These discrepancies surrounding the safety, postoperative outcomes, and pain management of TKAs/THAs are found throughout the literature and necessitate further research in order to provide patients with accurate information about cannabis use.

Limitations of this study include the retrospective case-series design at a single institution and the inability to determine the quantity or route of cannabis consumption. Furthermore, many patients were lost to follow-up, resulting in a low sample size and low total incidence of complications to use for comparison between groups. No criteria for comorbid conditions such as the Charlson comorbidity index were used due to incomplete patient data within their respective medical records; however, patients were matched based on age, sex, and BMI to limit any confounding factors. Additional trials with prospective, multicenter designs or a large database would be ideal for examining the postoperative effects and safety profiles of cannabis use and long-term clinical outcomes.

## Conclusions

The present study suggests that cannabis use in patients receiving TKAs or THAs does not correlate with an increased risk of post-operative PROMIS scores, hospital stay, complication rate, revision rate, or pain compared to matched controls. The literature on the postoperative complications in patients using cannabis is unclear regarding if cannabis is harmful or even beneficial after arthroplasty. With increased legalization efforts and the overall prevalence of cannabis use, more research, including randomized prospective trials, should be pursued to better guide management decisions in consideration for arthroplasty procedures.
